# Phytostabilization of Cd and Pb in Highly Polluted Farmland Soils Using Ramie and Amendments

**DOI:** 10.3390/ijerph17051661

**Published:** 2020-03-04

**Authors:** Mo-Ming Lan, Chong Liu, Shi-Jiao Liu, Rong-Liang Qiu, Ye-Tao Tang

**Affiliations:** 1 School of Environmental Science and Engineering, Sun Yat-sen University, Guangzhou 510275, China; lanmm@mail2.sysu.edu.cn (M.-M.L.); Liuch289@mail2.sysu.edu.cn (C.L.); Liushj39@mail2.sysu.edu.cn (S.-J.L.); eesqrl@mail.sysu.edu.cn (R.-L.Q.); 2 Guangdong Provincial Key Laboratory of Environmental Pollution Control and Remediation Technology, Sun Yat-sen University, Guangzhou 510275, China; 3 Guangdong Provincial Engineering Research Center for Heavy Metal Contaminated Soil Remediation, Sun Yat-sen University, Guangzhou 510275, China

**Keywords:** phytostabilization, ramie, soil amendments, Cd, Pb

## Abstract

In-situ remediation of heavy-metal-contaminated soil in farmland using phytostabilization combined with soil amendments is a low-cost and effective technology for soil pollution remediation. In this study, coconut shell biochar (CB, 0.1% and 0.5%), organic fertilizer (OF, 3.0%), and Fe-Si-Ca material (IS, 3.0%) were used to enhance the phytostabilization effect of ramie (*Boehmeria nivea* L.) on Cd and Pb in highly polluted soils collected at Dabaoshan (DB) and Yangshuo (YS) mine sites. Results showed that simultaneous application of CB, OF, and IS amendments (0.1% CB + 3.0% OF + 3.0% IS and 0.5% CB + 3.0% OF + 3.0% IS, DB-T5 and DB-T6) could significantly increase soil pH, reduce the concentrations of CaCl_2_-extractable Cd and Pb, and increase the contents of Ca, P, S, and Si in DB soil. Under these two treatments, the growth of ramie was significantly improved, its photosynthesis was enhanced, and its levels of Cd and Pb were reduced, in comparison with the control (DB-CK). After applying DB-T5 and DB-T6, the concentrations of Cd and Pb in roots were decreased by 97.7–100% and 64.6–77.9%, while in shoots they were decreased by up to 100% and 92.9–100%, respectively. In YS-T4 (0.5% CB + 3.0% OF), the concentrations of Cd and Pb in roots were decreased by 39.5% and 46.0%, and in shoots they were decreased by 44.7% and 88.3%. We posit that phytostabilization using ramie and amendments could reduce the Cd and Pb bioavailability in the soil mainly through rhizosphere immobilization and plant absorption. In summary, this study suggests that the use of tolerant plant ramie and simultaneous application of coconut shell biochar, organic fertilizer, and Fe-Si-Ca materials is an effective stabilization strategy that can reduce Cd and Pb availabilities in soil. Ultimately, this strategy may reduce the exposure risk of crops to heavy metal pollution in farmland.

## 1. Introduction

The 2014 National Soil Pollution Status Survey Bulletin of China [1] reported that the national soil over-standard rate for pollutants reached 16.1%, among which heavy metal pollution contributed most significantly (82% of the rate). In the meantime, the soil environmental quality of cultivated farmland is of high concern. The over-standard rate of arable land in the country exceeded 19.0%, including slight and light pollution (16.5%), moderate pollution (1.8%), and severe pollution (1.1%). A few studies have emphasized that heavy metal pollution in soils around mining, metallurgy, and other related industries is prominent, especially in the soils around nonferrous metal mining areas of South China [2]. Heavy metals, such as Cd and Pb, are main pollutants that may pose risks to the health of local residents through the food chain and other means [3,4]. The exceeding rates of Cd and Pb pollution in Chinese soils were 7.0% and 1.5%, respectively [1]. Among them, the severe pollution points of Cd and Pb exceeded 0.5% and 0.1%, respectively [1]. Therefore, the pollution with heavy metals in farmland needs to be solved urgently.

In the past decades, physical, chemical, and biological remediation technologies have been commonly used for soil heavy metal pollution control. The physical and chemical remediation technologies commonly used in remediation of polluted soils include excavation, electrokinetic remediation, chemical leaching and chemical immobilization [5]. Among them, chemical immobilization, which involves the use of carbon-, lime-, sepiolite-, bentonite-, clay-mineral-, and silicate-based soil amendments, appears to be the most feasible technology for remediation of heavy-metal-contaminated farmland soil [6,7,8,9]. Phytoremediation, which involves many uses of plants, is considered an in-situ, eco-friendly, and cost-effective approach to achieve remediation of soil risks [10]. In terms of different pollution levels, appropriate strategies should be used to maximize the phytoremediation effect. Phytoextraction, which uses hyperaccumulator plants to remove heavy metals from soil, is suggested for remediation of slightly and moderately contaminated soils [11,12], while phytostabilization is thought to be practical for remediation of multi-metal highly contaminated soils [13,14].

Phytostabilization generally refers to a plant remediation method that fixes pollutants to reduce their biological and environmental hazards [15]. Phytostabilization can reduce the bioavailability and mobility of heavy metals in soil. The metals are fixed in the rhizosphere by plant root adsorption and soil physical stability [16]. In recent years, increasing research has attempted to use commercially available, metal-tolerant plants for phytostabilization. For instance, *Lolium perenne* L. and composite amendments have been used for remediation of Zn, Pb, and Cd mine sites and have achieved good remediation effects; after the amendments were applied, toxicity of heavy metals on the plants was mitigated and root-to-root translocation of heavy metals was reduced; thus, the vegetation cover on the polluted soil could be restored [17]. Previous studies have attempted to use 42 native plant species in northern Mexico for stable plant restoration; the results showed that five of the native species might have potential for phytostabilization of Nacozari tailings and surrounding soils [18]. Strong tolerance of plants to heavy metal stress is fundamental for its potential use of phytostabilization. Rhizosphere effect is a response of plants to heavy metal stress. Plants can regulate the activity of rhizosphere microorganisms in plants by secreting root exudates, thereby affecting the activity of heavy metals in the soil [19].

Soil amendments play an important role in assisting phytostabilization of highly polluted soil. Addition of organic and inorganic soil amendments can promote the fixation of the metal and can thus enhance the effect of stable plant restoration [13]. Contaminated soils can be modified using organic/inorganic amendments depending on the optimal growth conditions of the plant. For example, soil amendments, such as lime, steel slag, and fly ash, are often used to neutralize soil acidity and reduce the activity of metals in soil [20,21]. The application of organic fertilizer and biochar can supplement soil nutrients and improve soil fertility to enhance the colonization of plants [22]. Application of biochar on mine-contaminated soil can effectively increase soil pH, TC, and TN, reduce the toxic effects of heavy metals on plants, and enhance the effect of *Cassia alata* L. on the phytostabilization of heavy-metal-contaminated soil [23]. Biochar can significantly increase soil pH, promote plant growth, and reduce the level of Cd in plants; the mechanism may be that the pH increases the adsorption capacity of the soil for metal cations or that the heavy metals are adsorbed on the surface of the biochar [24].

Ramie (*Boehmeria nivea* L.), which is a perennial herb of Urticaceae family, is widely distributed in China. It can adapt to harsh living conditions, such as mining sites. Meanwhile, ramie is regarded as a fiber plant with potential economic value, and the replacement of traditional cropping system (e.g., rice) with ramie could be a practical strategy to avoid cultivation of edible crops, hence reducing the risk of heavy metals entering the food chain. In this study, a pot experiment was conducted using ramie and a series of amendments (i.e., coconut shell biochar, organic fertilizer, and Fe-Si-Ca material) to study the phytostabilization effect of Cd- and Pb-polluted farmland soils collected at Dabaoshan (DB) and Yangshuo (YS) mining areas. The results are expected to provide theoretical and practical basis for establishing an effective remediation system for the multi-metal polluted farmland soil.

## 2. Materials and Methods

### 2.1. Preparation of Experimental Materials

#### 2.1.1. Soil Samples

Surface (0–20 cm) soil samples were collected from farmlands around DB multi-metal mine in Shang-ba Village, Guangdong Province, China (22.55° N and 113.72° E) and YS Pb/Zn mine in Si-di Village, Guangxi Zhuang Autonomous Region, China (25.02° N and 110.38° E) (Table 1). Two hundred kilograms of soil was collected at each of the two sampling points (DB and YS). DB mine is a large-scale polymetallic mine, and its surrounding farmland soils have been severely polluted by Pb, Cd, Zn, and Cu due to long-term mining activities in the past decades [3]. The soil of nearly 100 ha of farmland in YS Si-di Village has been polluted by Pb, Zn, Cd, and Cu through irrigation and flooding due to the unreasonable utilization of mineral resources, and the heavy metal content in agricultural products also exceeds the standard [4]. In terms of heavy metal content, DB and YS soils have similar Cd concentrations but differed substantially in the concentrations of Pb, Zn, and Cu.

#### 2.1.2. Plant and Soil Amendments

In the pot experiment, seedlings of a variety of ramie (Zhongsizhu No. 1, one year age) were purchased from the Institute of Bast Fiber Crops, Chinese Academy of Agricultural Sciences. Coconut shell biochar (CB) was produced by Hainan University, CB was made by putting the dried coconut shell in a muffle furnace and carbonizing it at 700 °C for 4 h [25]. Organic fertilizer (OF hereinafter) was produced from the mushroom residue, peanut bran, and bone meal by Nongfengbao Company. The OF contained organic matter ≥40%. Fe-Si-Ca material (IS hereinafter) was bought from Guangdong Province Shaoguan Steel Group Company Limited. It is a slag-like synthetic material [26] (Table 2).

### 2.2. Experimental Design

#### 2.2.1. Soil Simulation Experiment

Soil amendments were evenly added into a pot (26.8 cm × 26.0 cm × 17.8 cm). Each pot contained 5 kg of soil collected from DB and YS, respectively. The soil was stabilized after entering the soil amendments for 30 days. The experimental design consisted of 12 treatments, each with three replicates (Table 3). DB soil simulation experiment was mainly designed by comparing different application ratios of CB (0.1% and 0.5%) and different effects after adding OF (3.0%) and IS (3.0%). The YS soil simulation experiment mainly compared the different application rates of CB and the different effects with or without the addition of. As the YS soil has a relatively higher soil pH compared to DB soil, no alkaline material (IS) was added in the YS treatment.

#### 2.2.2. Pot Experiment

After the annual seedlings were transplanted, the seedlings of ramie (*Boehmeria nivea* L.) were grown in the pots (26.8 cm × 26.0 cm × 17.8 cm) with 5 kg soil from DB and YS for 90 days. The experimental design consisted of 12 treatments, each with three replicates. The treatment of the soil amendments was the same as the design of the soil simulation experiment (Table 3), and ramie was transplanted on the amended and stabilized soil. Two pot experiments were cultivated in an artificial climate chamber (AGC-D002Z) under the following conditions: 16 h light per day, illuminated and nonilluminated temperatures of 26 °C and 20 °C, respectively, and 60% humidity.

### 2.3. Sample Collection and Analysis

#### 2.3.1. Preparation of Soil and Plant Samples

The soil samples for simulation experiment were collected from the pot after the soil amendments were stabilized for 30 days. The rhizosphere soil for the pot experiment was collected using shaking root method [27]. The bulk soil samples were collected from the soil samples after the plant samples were collected and mixed. Plants were harvested on the day after root exudates were collected. Roots and shoots were separated and washed with deionized water, weighed, and dried [28].

#### 2.3.2. Chemical Analysis of Soil and Plant Samples

The soil and soil amendments pH was measured using a pH meter (LE438 pH, Mettler Toledo, Switzerland) in H_2_O (1:2.5, soil:solution ratio, dry *w*/*v*). The soil samples and soil amendments were dried thoroughly and then sieved through a 2 mm sieve and a 0.85 mm sieve. The 2 mm sieved soil samples and soil amendments were digested with HNO_3_ and HCl, and the digests were diluted to 25 mL with distilled water and filtered. The total concentrations of Cd, Pb, Cu, Zn, Ni, P, Ca, Fe, and S were determined by ICP-OES (Optima 5300DV, Perkin-Elmer, Waltham, MA, USA) and ICP-MS (Optima 356DV, Perkin-Elmer, Waltham, MA, USA). The CaCl_2_ extraction of the metals involved shaking 2.000 g of 0.85 mm sieved soil and soil amendments for 1.5 h with 20 mL 0.01 mol L^−1^ CaCl_2_, and the suspension was centrifuged for 10 min at 5000 rpm and filtered. The total concentrations of Cd, Pb, Cu, Zn, Ni, P, Ca, Fe, and S in plant samples were determined by ICP-OES and ICP-MS. The photosynthetic parameters of plants were measured by a portable photosynthetic measurement system (LI-6400XT, LI-COR, Lincoln, NE, USA).

### 2.4. Statistical Analysis

All data presented are means ± SD (standard deviation) of three independent replicates. Data were analyzed using analysis of variance. Means of significant difference were separated by *t* test or Tukey’s multiple range tests at *p* < 0.05.

## 3. Results and Discussion

### 3.1. Effects of Soil Amendments on Soil pH and Availability of Cd and Pb

**Figure 1:**
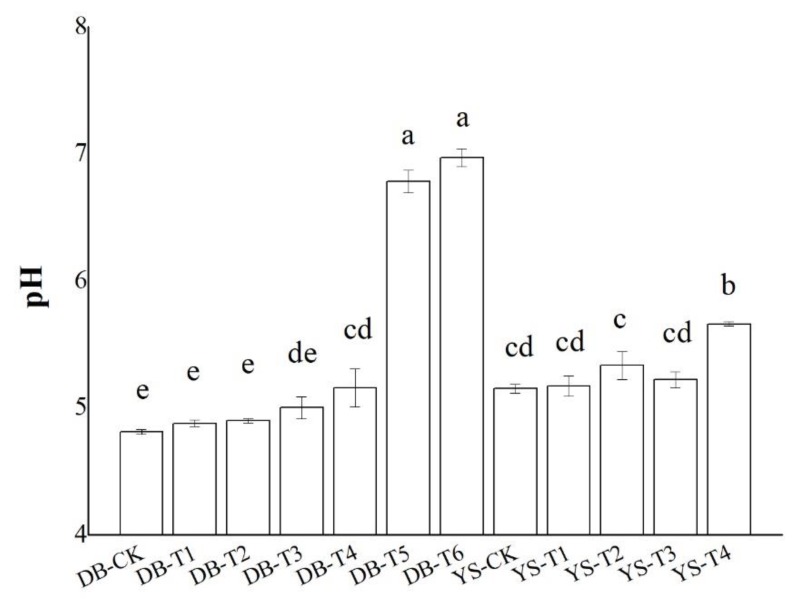
Effect of soil amendments on pH values in DB and YS soils. Means of significant difference are statistically analyzed by *t* test or Tukey’s multiple range tests at *p* < 0.05. The different lowercase letters in the figure indicate the significant difference in the soil pH values at different treatments.

The changes in the pH values of soil (DB and YS) after the application of soil amendments for 30 days are given in Figure 1. Soil amendments significantly increased soil pH by up to 2.2 units when 3.0% OF, 3.0% IS, and 0.5% CB (DB-T6) were added simultaneously. Soil pH was raised by 0.48 units when 0.5% CB and 3.0% OF (YS-T4) were added. The soil pH (4.8) of the DB soil and the soil pH (5.2) of the YS soil were low. The high pH value in the DB-T5 and DB-T6 soils might be ascribed to the addition of IS, which is strong in its basicity (Figure 1). Thus, the effect of improving pH was significant.

**Figure 2:**
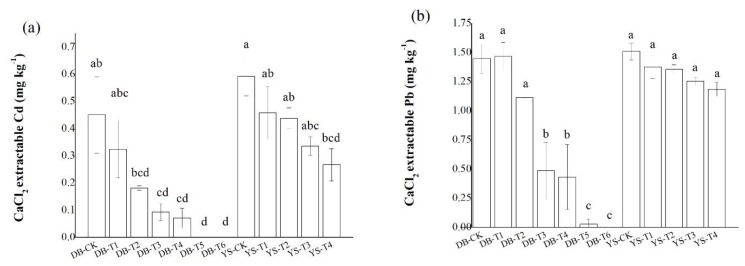
Effect of soil amendments on CaCl_2_-extractable (**a**) Cd and (**b**) Pb in DB and YS soils. Means of significant difference are statistically analyzed by *t* test or Tukey’s multiple range tests at *p* < 0.05. The different lowercase letters in the figure indicate the significant difference between the soil CaCl_2_-extractable Cd or Pb at different treatments.

Concentrations of CaCl_2_-extractable Cd/Pb in the DB-T6 soil were undetectable, significantly lower than those of the DB-CK (Figure 2). In YS-T4 soil, the CaCl_2_-extractable Cd and Pb concentrations were decreased by 54.9% and 21.5%, respectively, compared with those in YS-CK. Application of CB and OF among the two soils showed that the former had a greater reduction in CaCl_2_-extractable Pb content in soil than the latter. The changes in Cd and Pb availability in soil after applying the amendments might be related to the following factors. First, related studies (e.g., [25]) have shown that the adsorption of Pb^2+^ by CB depended on its CEC and ash content. The ash enhanced the electrostatic adsorption of the negative charge on the surface of the CB and increased the adsorption of Pb^2+^. In addition, a high CEC indicated a large surface charge number of the CB, which corresponded to a strong electrostatic adsorption capacity for metal cations. Second, the addition of OF should adjust the pH value of the soil, and the functional groups in the organic matter have a high affinity for Cd [29,30]. Other studies have found that high temperature can affect the biochar’s porosity development, and its pore parameters, like CEC, can significantly affect the chemical surface adsorption of biochar on heavy metals [31]. Soil pH is considered to be a key factor in determining Pb availability [32]. Moreover, metal cations mainly reduced their availability in soil by adsorption or formation of metal hydroxide precipitates when pH > 7.0 [33]. Third, the various elements in IS made it capable of precipitating or co-precipitating many metal ions, and such a system had a specific chemisorption potential for various metal ions [34]. The decrease in soil available Cd and Pb contents by applying IS might be caused by the increase in soil pH and available silicon content [34]. Our previous studies have shown that application of IS not only enhances the adsorption of heavy metals on its surface, but also precipitates heavy metals with Si, thereby reducing the bioavailability of heavy metals [27,35].

After the application of DB-T6, the contents of Ca, P, Si, and S in the soil increased significantly compared with those in DB-CK (Table S1, Supplementary Materials). Under DB-T6 treatment, the total amount of Ca in the soil increased by more than 10 times, and the total amount of Si increased by nearly 2 times (Table S1). The reason for the increase in nutrient content in soil was probably due to the application of soil amendments, which could improve soil fertility and supplement some nutrients in soil [27,36,37,38]. Increasing P content in soil increased pyromorphites formation and Pb-phosphate formation may be one of the reasons for reducing the effective state of Pb in soil [39].

### 3.2. Effects of Soil Amendments on Plant Growth and Photosynthesis

A significant difference was observed in fresh weight and plant height among treatments and CK (Table 4). Compared with DB-CK, the root biomass of DB-T6 increased significantly by 43.2%, shoot biomass increased significantly by 47.8%, and plant height increased by 19.4%. However, the difference between root and shoot biomass of YS treatments (T1–T4) and YS-CK was not significant. After 90 days of cultivation, the photosynthetic parameters were evidently changed among the soil treatments and the control. All treatments with soil amendments caused a significant increase in photosynthetic rate, DB-T6 and YS-T4 have the most significant effects. Conversely, transpiration rate was significantly reduced after the application of the soil amendments between the two pot experiments. In DB-T6, the net photosynthetic rate of plants was more than twice that in DB-CK.

The results that DB-T5 and DB-T6 to which IS was applied had the best effect on promoting plant biomass while YS-T4 had no significant effect may be due to the fact that the soil amendments supplemented some nutrients needed for plant growth. The use of the soil amendments increased the content of soil nutrient elements (P, Ca, S, and Si, Table S1). The promotion effect of DB-T6 on plant biomass may be because the application of alkaline amendment (i.e., IS) on acid soil has a significant effect on improving soil pH and provides a suitable acid–base environment for plants. However, the application of CB or CB plus OF on the improvement of plant growth conditions is still insufficient. Therefore, rationally applying the soil amendments not only can provide plants with nutrients but also can reduce the activity of Cd and Pb in the soil, thereby promoting plant growth and enhancing plant photosynthesis [40,41]. Under Cd stress, factors such as plant photosynthetic enzyme activity, photosynthetic pigment content, and leaf stomatal conductance were negatively affected; thus, the photosynthesis was impaired [42]. For DB-T6, an increase in the total amount of Ca and Si in soil (Table S1) might lead to an increase in the bioavailability of Ca and Si in the soil. Studies have shown that Si application could increase the accumulation of SiO_2_ in the epidermal layer of plant leaves, which was conducive to enhancing the strength of the leaves and erecting the leaves [43]. Ca was involved in photosynthesis processes, such as photosynthetic electron flow and light-dependent metabolic reactions [44,45]. In the Cd-contaminated medium, the application of Ca could restore the photosynthesis of plants under Cd stress [46]. This deduction was consistent with the results of the current experiment.

### 3.3. Effects of Soil Amendments on Plant Heavy Metal Accumulation

**Figure 3:**
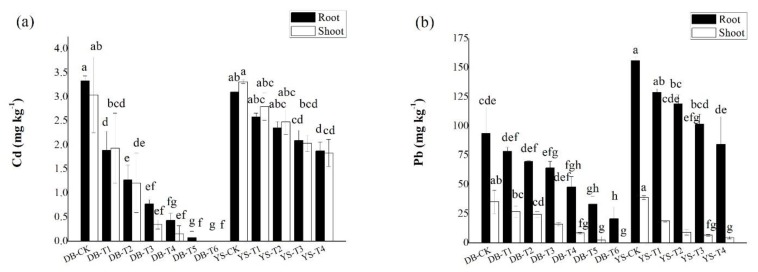
Concentrations of (**a**) Cd and (**b**) Pb in roots and shoots of ramie. Means of significant difference are statistically analyzed by *t* test or Tukey’s multiple range tests at *p* < 0.05. The different lowercase letters in the figure indicate the significant difference between the Cd and Pb of root or shoot of ramie at different treatments.

The concentrations Cd/Pb in the roots of ramie were generally higher than those in the shoots (Figure 3). For DB, the Cd and Pb concentrations in roots of DB-T5 and DB-T6 were decreased by 97.7–100% and 64.6–77.9% compared with those in the DB-CK. The shoot Cd and Pb concentrations of DB-T5 and DB-T6 were decreased by up to 100% and by 92.9–100% compared with the DB-CK. For YS, analogously, YS-T4 treatment reduced 39.5% Cd and 46.0% Pb in the roots compared with the YS-CK. The shoot Cd/Pb concentrations of YS-T4 were decreased 44.7% and 88.3%, respectively. For Cd in plants (especially in YS), the difference between root and shoot was not obvious. In other studies, it has also been found that after Cd pollution increases, the concentration of Cd accumulated in the shoots of ramie will gradually increase, and the difference of Cd concentration between shoots and roots gradually becomes smaller [47,48]. Previous studies have shown that ramie has a strong tolerance to Cd in Cd-contaminated soils, which could limit the mobility of Cd after entering the plant root [49]. The complexation of Cd with sulfur-rich peptides and organic ligands, such as organic acids, and the storage of Cd by vacuoles are the main mechanisms of tolerance of some plants to Cd stress [50]. Data have shown [50] that more than 50% of Cd is accumulated in plant cell walls. The cell wall contains polysaccharides and proteins, and the negatively charged sites on the surface bind to Cd; as a result, the mobility of Cd^2+^ in the plant becomes limited. After the increase in Pb pollution, the response strategy of hemp to Pb stress was mainly manifested in that Pb mainly accumulated in plant roots and remained in the cell wall of the root system in addition to the exclusion of Pb from shoots [51].

### 3.4. Effects of Soil Amendments on Rhizosphere Immobilization of Cd/Pb

**Figure 4:**
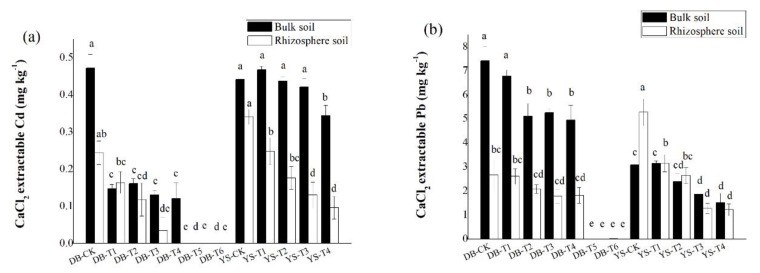
Difference in CaCl_2_-extractable (**a**) Cd and (**b**) Pb of rhizosphere and bulk soils in DB and YS. Means of significant difference are statistically analyzed by *t* test or Tukey’s multiple range tests at *p* < 0.05. The different lowercase letters in the figure indicate the significant difference between the CaCl_2_-extractable Cd and Pb of rhizosphere or nonrhizosphere soils at different treatments.

**Figure 5:**
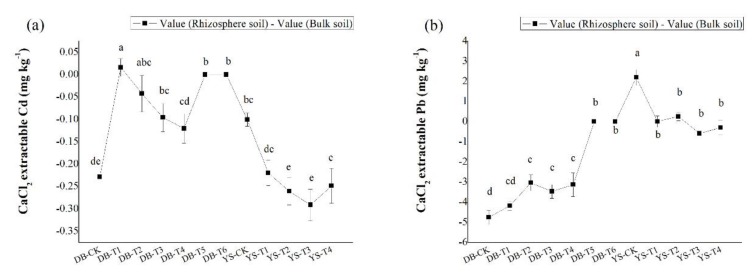
Concentrations of CaCl_2_-extractable (**a**) Cd and (**b**) Pb of bulk and rhizosphere soils in DB and YS. Means of significant difference are statistically analyzed by *t* test or Tukey’s multiple range tests at *p* < 0.05. The different lowercase letters in the figure indicate the significant difference between the rhizosphere soil CaCl_2_-extractable Cd and Pb at different treatments, and the different uppercase letters indicate significant differences between treatments under the bulk soil CaCl_2_-extractable Cd and Pb.

Compared with the control, the CaCl_2_-extractable Cd/Pb concentrations in bulk soil showed a significant decrease with the application dosage of amendments (Figure 4), consistent with the trend in the soil simulation experiment (Figure 2). Under the DB control treatment, the concentrations of CaCl_2_-extractable Cd/Pb in the rhizosphere soil were 48.3% and 64.2% lower than those in the bulk soil, respectively, which could be ascribed to the plant effect. The available Cd/Pb can be absorbed onto root surface and assimilated into roots, thereby reducing the bioavailabities of Cd/Pb in the soil [52]. After applying the soil amendment, the concentrations of CaCl_2_-extractable Cd (DB-T4) and CaCl_2_-extractable Pb (DB-T1 to DB-T4) in the rhizosphere were still lower than in the bulk soil (Figure 5), but their differences were less than those of the control (Figure 5), indicating that the decrease of the bioavailable Cd (DB-T4) and bioavailable Pb (DB-T1 to DB-T4) results from the combination effect of root absorption and immobilization by soil amendments. After the application of the alkaline soil amendment IS (DB-T5 and DB-T6), the concentration of CaCl_2_-extractable Cd/Pb in the rhizosphere was close to zero, which had no difference with the bulk soils. This is due to the strong immobilization of IS application, as shown in the simulation experiment (Figure 2). In YS control soil, the CaCl_2_-extractable Cd concentration in the bulk soil was significantly higher than that of rhizosphere soil (Figure 5). The CaCl_2_-extractable Cd gradually decreased when the soil amendments were applied (Figure 4), but the differences between bulk soil and rhizosphere soil gradually increased (YS-T1 to YS-T3) (Figure 5). It is speculated that the addition of organic fertilizer may enhance the absorption capacity of plant roots [53]. The concentrations of CaCl_2_-extractable Pb in the rhizosphere soil of control (YS-CK) was much higher than that in bulk soil (Figure 4 and Figure 5), indicating that the rhizosphere exudates may have activated the heavy metals such as Pb [54,55,56,57,58]. The difference in the concentrations of Pb between rhizosphere and bulk soil was not significant at YS-T1 to YS-T4 (Figure 5), which suggests that soil amendments (CB and OF) contribute more to the immobilization of Pb in soil than plant absorption of this metal.

## 4. Conclusions

This study explored the effects of applying ramie, a heavy-metal-tolerant economic plant, with soil amendments (i.e., CB, OF, and Fe-Si-Ca materials) on the phytostabilization of Cd and Pb highly polluted soil collected from DB and YS mine areas. The results showed that simultaneous application of CB, OF, and Fe-Si-Ca could significantly increase soil pH and reduce more than 90.0% of CaCl_2_-extratable Cd and Pb in soil. The three amendments could significantly increase the content of nutrients, such as Ca, P, S, and Si, in soil. In addition, the combination of the three amendments could enhance the growth of ramie on heavily polluted soils, increase its photosynthesis, and reduce more than 60.0% the accumulation of Cd and Pb in ramie tissues. In summary, the application of ramie with CB, OF, and Fe-Si-Ca amendments is an effective phytostabilization technology for remediating Cd- and Pb-contaminated soil, reducing the potential risk to surrounding environment. Further research is still needed to monitor the long-term effects of phytostabilization of heavy metals and the change of soil quality after the application of the ramie plant together with soil amendments in the field.

## Figures and Tables

**Table 1:** Physical and chemical properties of the experimental soil.

Soil Sample	Soil Type	pH	Total Concentration (mg kg^−1^)					CaCl_2_-Extractable (mg kg^−1^)	
			Cd	Pb	Cu	Zn	Ni	Cd	Pb
DB	Paddy soil	4.8 ± 0.02	1.26 ± 0.87	681 ± 48.31	343 ± 9.23	339 ± 11.5	7.04 ± 1.56	0.45 ± 0.14	1.45 ± 0.13
YS	Paddy soil	5.2 ± 0.04	1.29 ± 0.43	454 ± 5.56	75.1 ± 0.75	768 ± 5.30	14.4 ± 0.17	0.59 ± 0.07	1.51 ± 0.07

Physical and chemical properties of the experimental soil. All data presented are means ± SD (standard deviation) of three independent replicates. DB: Dabaoshan, YS: Yangshuo. The methodologies for measuring the properties of the soils are given in Section 2.3.2.

**Table 2:** Physical and chemical properties of the soil amendments.

Amendment Type	pH	Total Concentration (mg kg^−1^)				Total Concentration (%)			CaCl_2_-Extractable (mg kg^−1^)	
		Cd	Pb	S	Si	P	Ca	Fe	Cd	Pb
CB	7.8	<LOD	<LOD	484 ± 31.2 ^c^	14.4 ± 4.12 ^c^	0.15% ^c^	0.36% ^c^	0.66% ^b^	<LOD	<LOD
OF	10.0	<LOD	<LOD	7.19 × 10^3^ ± 437 ^a^	43.9 ± 1.57 ^b^	1.08% ^b^	2.77% ^b^	0.74% ^a^	<LOD	<LOD
IS	12.4	<LOD	<LOD	1.11 × 10^3^ ± 117 ^b^	74.9 ± 6.45 ^a^	0.57% ^a^	16.2% ^a^	10.2% ^a^	<LOD	<LOD

All data presented are means ± SD (standard deviation) of three independent replicates. Means of significant difference are statistically analyzed by *t* test or Tukey’s multiple range tests at *p* < 0.05. “<LOD” represents metal concentration below the detection limit. Total Cd (LOD < 0.1 mg kg^−1^), Total Pb (LOD < 1.4 mg kg^−1^), CaCl_2_-extractable Cd (LOD < 0.01 mg kg^−1^), CaCl_2_-extractable Pb (LOD < 0.2 mg kg^−1^). The different superscript lowercase letters in the table indicate the significant difference in the nutrient elements (S, Si, P, Ca, Fe) at different soil amendments. CB: Coconut shell biochar, OF: Organic fertilizer, IS: Fe-Si-Ca materials. The methodologies for measuring the properties of the amendments are given in the Section 2.3.2.

**Table 3:** Soil simulation experiment scheme.

Number	Description
DB-CK	Control, Soil DB
DB-T1	Soil DB + 0.1% CB
DB-T2	Soil DB + 0.5% CB
DB-T3	Soil DB + 0.1% CB + 3.0% OF
DB-T4	Soil DB + 0.5% CB + 3.0% OF
DB-T5	Soil DB + 0.1% CB + 3.0% OF + 3.0% IS
DB-T6	Soil DB + 0.5% CB + 3.0% OF + 3.0% IS
YS-CK	Control, Soil YS
YS-T1	Soil YS + 0.1% CB
YS-T2	Soil YS + 0.5% CB
YS-T3	Soil YS + 0.1% CB + 3.0% OF
YS-T4	Soil YS + 0.5% CB + 3.0% OF

Soil simulation experiment scheme.

**Table 4:** Photosynthetic and biomass parameters in Dabaoshan (DB) and Yangshuo (YS) pot experiments.

Treatment	Photosynthesis Rate (μmol m^−2^ s^−1^)	Transpiration Rate (μmol m^−2^ s^−1^)	Shoot Biomass (Fw, g/plant)	Root Biomass (Fw, g/plant)	Height (cm)
DB-CK	0.35 ± 0.03 ^e,f^	0.83 ± 0.04 ^d^	96.5 ± 15.1 ^b^	41.2 ± 1.95 ^b,c^	53.0 ± 7.6 ^a,b,c^
DB-T1	0.50 ± 0.06 ^d,e^	0.64 ± 0.01 ^e,f^	79.0 ± 11.7 ^b^	35.0 ± 3.75 ^c^	54.3 ± 3.2 ^a,b,c^
DB-T2	0.53 ± 0.11 ^c,d,e^	0.71 ± 0.01 ^d,e^	92.8 ± 17.6 ^b^	39.3 ± 8.31 ^b,c^	54.3 ± 6.8 ^a,b,c^
DB-T3	0.59 ± 0.05 ^b,c,d^	0.53 ± 0.05 ^f,g^	88.4 ± 1.27 ^b^	42.4 ± 3.56 ^b,c^	55.3 ± 7.5 ^a,b,c^
DB-T4	0.70 ± 0.03 ^b,c^	0.48 ± 0.01 ^g^	90.7 ± 2.32 ^b^	39.6 ± 6.48 ^b,c^	56.7 ± 4.5 ^a,b,c^
DB-T5	0.73 ± 0.18 ^b^	0.48 ± 0.04 ^g^	115 ± 14.1 ^a,b^	53.5 ± 2.48 ^a,b^	59.7 ± 8.6 ^a,b^
DB-T6	1.13 ± 0.04 ^a^	0.78 ± 0.01 ^d,e^	143 ± 2.15 ^a^	59.1 ± 2.23 ^a^	63.3 ± 5.7 ^a^
YS-CK	0.28 ± 0.02 ^f^	1.45 ± 0.01 ^a^	95.4 ± 8.14 ^b^	35.1 ± 7.05 ^b^	40.3 ± 8.7 ^c^
YS-T1	0.56 ± 0.01 ^c,d,e^	1.30 ± 0.08 ^a,b^	99.4 ± 4.25 ^b^	42.7 ± 6.03 ^b^	40.7 ± 4.2 ^c^
YS-T2	0.56 ± 0.02 ^c,d,e^	1.14 ± 0.05 ^c^	105 ± 32.6 ^a,b^	37.8 ± 4.00 ^a,b^	40.3 ± 3.8 ^c^
YS-T3	0.70 ± 0.02 ^b,c^	1.23 ± 0.05 ^b,c^	100 ± 6.18 ^b^	34.6 ± 3.50 ^b^	43.3 ± 2.5 ^b,c^
YS-T4	1.11 ± 0.17 ^a^	1.30 ± 0.13 ^a,b^	102 ± 6.99 ^b^	39.2 ± 4.81 ^b^	48.3 ±3.2 ^a,b,c^

Photosynthetic and biomass parameters in Dabaoshan and Yangshuo pot experiments. All data presented are means ± SD (standard deviation) of independent replicates. Values in the table are mean (*𝑛* = 3). Means of significant difference are statistically analyzed by *t* test or Tukey’s multiple range tests at *p* < 0.05. The different lowercase letters in the table indicate the significant difference in the photosynthetic and biomass parameters at different treatments.

## Supplementary Materials

The following are available online at https://www.mdpi.com/1660-4601/17/5/1661/s1, Table S1: Nutrient element contents in soil of Dabaoshan (DB) and Yangshuo (YS) mining areas (mg kg^−1^).
