# Supplementary material for: Phytostabilization of Cd and Pb in Highly Polluted Farmland Soils Using Ramie and Amendments
Source: Int J Environ Res Public Health. 2020 Mar 4;17(5):1661. doi: 10.3390/ijerph17051661 (PMC7084681; doi:10.3390/ijerph17051661)
Supplement: Supplementary file 1 [file ijerph-17-01661-s001.pdf]

Supporting Information:

Table 1. Nutrient elements contents in soil of Dabaoshan(DB) and Yangshuo(YS) mining area (mg kg^{-1}).

Treatment	P	Ca	Fe	S	Si
DBCK	1202.76±31.42c	1224.31±47.18b	58379.22±4112.77a	1051.22±33.25c	72.27±19.67e
DB0.1CB	1193.08±3.26c	1238.87±34.82b	59837.39±607.97a	1151.17±18.07b	106.85±19.79ef
DB0.5CB	1172.40±19.12c	1282.57±28.24b	58225.44±4662.92a	1156.43±53.09b	99.77±18.72ef
DB0.1CB3.0OF	1469.97±133.03b	1954.37±194.80b	58895.23±803.82a	1153.63±6.58b	131.88±18.55de
DB0.5CB3.0OF	1430.58±80.37b	1861.07±112.52b	60231.49±1134.93a	1188.48±56.79ab	139.15±26.33cde
DB0.1CB3.0OF3.0SG	1672.28±172.44a	12120.78±7312.32a	59223.80±2720.82a	1238.41±65.54a	188.05±44.65ab
DB0.5CB3.0OF3.0SG	1774.21±97.86a	14440.16±5794.54a	58262.15±2174.50a	1254.47±42.00a	206.59±46.05a
YSCK	634.27±17.08e	1224.31±47.18b	23814.94±554.37b	1182.91±33.70f	156.68±41.17ef
YS0.1CB	688.61±79.38e	1238.87±34.82b	23923.08±698.01b	1131.77±45.61f	92.69±39.77f
YS0.5CB	631.25±38.46e	1282.57±28.24b	23669.61±887.38b	1100.44±87.05f	169.98±84.63bcde
YS0.1CB3.0OF	886.21±54.38d	1954.37±194.80b	23835.7±547.78b	1249.64±85.28d	160.65±73.95abcd
YS0.5CB3.0OF	888.68±15.53d	1861.07±112.52b	23196.18±355.32b	1188.48±56.79d	124.79±31.55abc

Table S1. Nutrient element contents in soil of Dabaoshan (DB) and Yangshuo (YS) mining areas (mg kg^{-1}). All data presented are means ± SD (standard deviation) of independent replicates. Values in the graph are mean (n = 3). Means of significant difference are statistically analyzed by t test or Duncan's multiple range tests at P<0.05.

Table 2. Nutrient elements contents in shoots of ramie (mg kg^{-1}).

Treatment	P	Ca	Fe	S	Si
DBCK	835.31±87.69f	6628.83±1603.73e	4976.29±1153.63def	1062.35±238.57bc	107.05±8.67e
DB0.1CB	886.52±42.09ef	9510.12±586.40bcde	6310.02±1000.79de	1448.81±175.37bc	111.67±1.98de
DB0.5CB	970.16±133.47def	9470.82±1451.56bcde	6688.43±686.01cd	1278.19±137.41bc	115.96±6.11cde
DB0.1CB3.0OF	1032.82±56.82cdef	10614.34±1067.41bcd	7222.11±706.65bcd	1511.75±588.43bc	117.53±0.85cde
cdeDB0.5CB3.0OF	1052.52±97.24cdef	11558.65±969.32b	8913.41±800.63abc	1814.85±379.82b	119.08±7.52cde
DB0.1CB3.0OF3.0SG	1338.08±230.94bcdef	15499.57±3054.24a	9019.71±1635.65ab	3670.64±682.80a	139.84±6.68ab
DB0.5CB3.0OF3.0SG	1461.01±504.68bcd	15578.85±3927.24a	10635.48±1597.11a	3388.94±942.46a	143.60±10.52a
YSCK	1375.64±0.63bcde	7400.26±385.32de	2984.48±220.10f	792.35±74.23c	86.55±6.47f
YS0.1CB	1554.30±190.93bc	8276.97±1043.97cde	3806.20±1080.33f	880.88±42.36c	104.15±9.86e
YS0.5CB	1665.17±148.66b	9096.95±736.68bcde	4352.72±972.94ef	882.58±52.68c	106.58±7.58e
YS0.1CB3.0OF	2156.73±389.44a	11406.95±1039.28bc	6996.59±1986.72bcd	1155.68±116.68bc	124.68±14.14cd
YS0.5CB3.0OF	2244.47±597.78a	12316.99±642.22b	7037.29±1534.71bcd	1223.26±19.22bc	128.38±8.56bc

Table S2. Nutrient element contents in shoots of ramie. All data presented are means ± SD (standard deviation) of independent replicates. Values in the graph are mean (n = 3). Means of significant difference are statistically analyzed by t test or Duncan's multiple range tests at P<0.05.

Table 3. Nutrient elements contents in roots of ramie (mg kg^{-1}).

Treatment	P	Ca	Fe	S	Si
DBCK	835.31±87.69f	6628.83±1603.73e	4976.29±1153.63def	1062.35±238.57bc	107.05±8.67e
DB0.1CB	886.52±42.09ef	9510.12±586.40bcde	6310.02±1000.79de	1448.81±175.37bc	111.67±1.98de

DB0.5CB	970.16±133.47def	9470.82±1451.56bcde	6688.43±686.01cd	1278.19±137.41bc	115.96±6.11cde
DB0.1CB3.0OF	1032.82±56.82cdef	10614.34±1067.41bcd	7222.11±706.65cde	1511.75±588.43bc	117.53±0.85cde
DB0.5CB3.0OF	1052.51±97.24cdef	11558.65±969.32b	8913.41±800.63abc	1814.85±379.82b	119.08±7.52cde
DB0.1CB3.0OF3.0SG	1338.08±230.94bcdef	15499.57±3054.24a	9019.71±1635.65ab	3670.64±682.80a	139.84±6.68ab
DB0.5CB3.0OF3.0SG	1461.01±504.68bcd	15578.85±3927.31a	10635.48±1597.11a	3388.94±942.46a	143.60±10.52a
YSCK	1375.64±0.63bcde	7400.26±385.32de	2984.48±220.10f	792.35±74.23c	86.55±6.47f
YS0.1CB	1554.30±190.93bc	8276.97±1043.97cde	3806.20±1080.33f	880.88±42.36c	104.15±9.86e
YS0.5CB	1665.17±148.66b	9096.95±736.68bcde	4352.72±972.94ef	882.58±52.68c	106.58±7.58e
YS0.1CB3.0OF	2156.73±389.44a	11406.95±736.68bc	6996.59±1986.72cde	1155.68±116.68bc	124.68±14.14cd
YS0.5CB3.0OF	2244.47±597.78a	12316.99±642.22b	7037.29±1534.71cde	1223.26±19.22bc	128.38±8.56bc

Table S3. Nutrient element contents in shoots of ramie. All data presented are means ± SD (standard deviation) of independent replicates. Values in the graph are mean (n = 3). Means of significant difference are statistically analyzed by t test or Duncan's multiple range tests at P<0.05.
